# Developmental Prosopagnosia and Elastic Versus Static Face Recognition in an Incidental Learning Task

**DOI:** 10.3389/fpsyg.2020.02098

**Published:** 2020-08-31

**Authors:** Tom Bylemans, Leia Vrancken, Karl Verfaillie

**Affiliations:** Brain and Cognition, Faculty of Psychology and Educational Sciences, KU Leuven, Leuven, Belgium

**Keywords:** face perception, moving vs. static faces, developmental prosopagnosia, visual learning, incidental learning

## Abstract

Previous research on the beneficial effect of motion has postulated that learning a face in motion provides additional cues to recognition. Surprisingly, however, few studies have examined the beneficial effect of motion in an incidental learning task and developmental prosopagnosia (DP) even though such studies could provide more valuable information about everyday face recognition compared to the perception of static faces. In the current study, 18 young adults (Experiment 1) and five DPs and 10 age-matched controls (Experiment 2) participated in an incidental learning task during which both static and elastically moving unfamiliar faces were sequentially presented and were to be recognized in a delayed visual search task during which the faces could either keep their original presentation or switch (from static to elastically moving or vice versa). In Experiment 1, performance in the elastic-elastic condition reached a significant improvement relative to the elastic-static and static-elastic condition, however, no significant difference could be detected relative to the static-static condition. Except for higher scores in the elastic-elastic compared to the static-elastic condition in the age-matched group, no other significant differences were detected between conditions for both the DPs and the age-matched controls. The current study could not provide compelling evidence for a general beneficial effect of motion. Age-matched controls performed generally worse than DPs, which may potentially be explained by their higher rates of false alarms. Factors that could have influenced the results are discussed.

## Introduction

Faces are omnipresent visual stimuli that provide observers with crucial information in social interactions. In everyday life, faces alter every second with emotional states and functions, such as eating, talking, and looking (Barton, 2003; Krumhuber et al., 2013; Jack and Schyns, 2015). Given these real-life facial dynamics, it is somewhat surprising that most of the research on face perception has focused on static images of faces. This seems to be equally true for research with people who lack normal face recognition skills, known as prosopagnosia (Bodamer, 1947, first coined the term), even though research with moving faces in this population could provide more valuable information compared to research with static faces. The current study aimed to address these issues by employing a newly designed incidental learning task with elastically moving versus static faces, which were used to test a population of 18 normally developing observers in Experiment 1 and five developmental prosopagnosics (DPs) and 10 age-matched controls in Experiment 2.

In acquired prosopagnosia (AP) face recognition difficulties arise as a result of observable damage to face processing regions in the brain (e.g., Yin, 1970; Barton, 2008; Busigny et al., 2010; Van Belle et al., 2010b,c; Davis-Thompson et al., 2014; DeGutis et al., 2014). DP – also commonly referred to as congenital prosopagnosia (e.g., Behrmann and Avidan, 2005) – is a condition characterized by an impairment in face identification, which is believed to be present from birth or early in life, without observable brain damage, but with intact low-level visual acuity, intact socio-cognitive abilities, and normal intellectual abilities (e.g., Barton et al., 2003; Behrmann et al., 2005; Le Grand et al., 2006; Avidan and Behrmann, 2009; Nishimura et al., 2010; Stollhoff et al., 2011; Verfaillie et al., 2014; Shah et al., 2015; Cook and Biotti, 2016; Fisher et al., 2017; Gray et al., 2017). Some researchers have suggested a genetic component underlying the condition (e.g., Biotti and Cook, 2016; Cattaneo et al., 2016), and a prevalence of around 2.5% of the population has been reported (Kennerknecht et al., 2006, 2008). In the current study, participants with DP (in addition to control subjects) were tested.

A plethora of studies on underlying mechanisms to explain the face recognition deficit in DP have been conducted. However, results remain inconclusive. For example, several studies have revealed abnormal holistic processing in DP (e.g., Avidan et al., 2011; Liu and Behrmann, 2014), a processing style that enables human observers to perceive the face as a whole (i.e., all separate facial features are glued together to form a global image of the face), and which is sometimes believed to underlie rapid face recognition abilities in general (Maurer et al., 2002; Van Belle et al., 2010a; Rivolta, 2014; Vrancken et al., 2017, 2019). These studies suggest that difficulties to perceive faces as a whole in DP could elicit an overreliance on specific facial features (e.g., Palermo et al., 2011). In contrast, an elaborate study with eight DP participants revealed normal holistic processing (Le Grand et al., 2006), as observed by a preserved ability to integrate featural information into a global percept.

Furthermore, researchers have suggested that atypical gaze behavior toward the face, with fewer fixations on the eye-region, during development, may reflect an important contributing factor to DP (e.g., Schwarzer et al., 2007; also see Bobak et al., 2016). This finding seems to be supported by other studies (e.g., DeGutis et al., 2012b), but more research is needed before firm conclusions can be drawn (e.g., see Geskin and Behrmann, 2018; Peterson et al., 2019). For the time being, researchers can only agree that people with DP form a heterogeneous group characterized by variation in clinical profiles (Stollhoff et al., 2011; Susilo and Duchaine, 2013; Dalrymple and Duchaine, 2016; Bate and Tree, 2017).

Studies on the perception of moving faces are relatively sparse compared to the multitude of static face processing studies. Like other research on moving faces, the theoretical basis for the current study is provided by the beneficial effect of motion, evidenced by better recognition when moving faces are presented during a learning phase, compared to statically presented faces (e.g., O’Toole et al., 2002; Roark et al., 2003; Zebrowitz and Montepare, 2008; Thornton et al., 2011; Xiao et al., 2014; Butcher and Lander, 2017). Two influential hypotheses have been postulated to explain this beneficial effect. First, the supplemental information hypothesis (SIH) posits that in addition to the unchangeable structure of the face (e.g., two eyes above a nose), faces contain idiosyncratic movements, which can act as additional cues to identity during later recognition (Bruce and Valentine, 1988; Knight and Johnston, 1997; Lander et al., 1999, 2001; Lander and Bruce, 2000; Hill and Johnston, 2001; Knappmeyer et al., 2001; O’Toole et al., 2002; Butcher and Lander, 2017). Second, the representation enhancement hypothesis (REH) proposes that recognition by facial motion is facilitated by enhancing the three-dimensional structure of a face (Pike et al., 1997; Christie and Bruce, 1998; O’Toole et al., 2002). More specifically, motion elicits a more detailed view of the characteristic structure of a face, thereby providing more information of that structure compared to a statically perceived face. This facilitates later recognition of that face from memory.

Whether motion acts as a beneficial cue to face recognition in DP remains inconclusive. In one study, the role of motion on memory performance for faces in DP was examined, but only one of four patients showed improvement with motion (Longmore and Tree, 2013). The researchers stated, however, that because of the brief time during which several unfamiliar faces had to be learned, the memory task could have been too challenging, which might have reduced the possibility to find a beneficial effect of motion. They nonetheless concluded that DPs appear able to use characteristic facial motion as a supplemental cue to aid face processing (thus providing evidence for the SIH). Another study on the motion effect in DP found differing results when comparing elastic (or non-rigid) and rigid motion (Maguinnes and Newell, 2015). Elastic motion occurs when the face transforms in shape, while rigid motion occurs when the face moves but does not change in shape (Christie and Bruce, 1998; Girges et al., 2015). Maguinnes and Newell (2015) posited that rigid motion can benefit DPs ability to match faces for identity, while elastic motion can interfere with the representation of structural face information through changes in the internal features of the face. This is particularly difficult for DPs considering that some of them adopt a more feature-based matching strategy (e.g., Palermo et al., 2011). In a study by Steede et al. (2007), one DP participant was able to use facial motion for discriminating and learning identities. However, based on the findings from Maguinnes and Newell (2015), it can be assumed that this one DP was potentially employing a learning strategy that was focused on rigid motion (instead of non-rigid motion) as rigid motion does not interfere with the representation of structural information. This could be the reason why this DP showed preserved discrimination of moving faces. Finally, in a study implementing a famous face recognition task, DPs were able to use both elastically and rigidly moving faces to their advantage when matching identities, compared to static face images (Bennetts et al., 2015). These researchers concluded that DPs can learn the characteristic motion patterns for famous faces and can use them as a supplemental cue to aid recognition (SIH). However, they also explained their findings in terms of the REH, by positing that more views (30-frames per second) gave DPs more opportunities to match the faces to their stored representations.

Some remarks can be formulated regarding the investigations described above. First, the studies provided relevant insight by implementing moving faces as it serves to be a more ecologically valid approach. Indeed, many of the studies using static images of faces have obscured interpretation in terms of real (i.e., everyday) face perception by controlling for noise and eliminating variance in dimensions except for those which researchers wished to manipulate (Burton, 2013). Furthermore, several studies that implemented moving faces adopted an intentional learning task, meaning that the participants probably knew that face recognition would be tested later. In everyday life, however, humans rarely intentionally learn the faces of the people they encounter. Additionally, intentional learning can promote non-face related strategies by focusing on specific features (e.g., “this face has curly hair, I will try to remember the curly hair”). In the context of ecological validity, it is therefore preferred to use incidental learning paradigms. Second, most of the studies provided evidence for DPs’ ability to use characteristic facial motion as a cue to match or recognize faces (SIH). However, some of the researchers also reported evidence for the role of facial motion in support of the REH (Bennetts et al., 2015; Maguinnes and Newell, 2015). Still, the issue whether DPs benefit from seeing the 3D structure of a moving face remains largely inconclusive. Third, the inconsistency of the results could be due to the small number of reported cases and the prevalence of single case studies, an issue that was previously noted by Le Grand et al. (2006). In the studies described above, the number of DPs ranged from one (e.g., Steede et al., 2007) to nine (e.g., Bennetts et al., 2015). Finally, although in the delayed matching-to-sample studies the faces were never presented at the same time (Longmore and Tree, 2013; Experiment 2; Maguinnes and Newell, 2015), the interval between the to be learned face and the matching task was still brief and none of the studies used retention periods that lasted more than a few seconds. To our knowledge, only two studies tested recognition for moving faces using a delayed recognition paradigm (i.e., target faces were learned sequentially before recognition was tested; Longmore and Tree, 2013; Experiment 1; Steede et al., 2007). However, Steede et al. (2007) provided a series of learning phases during which participants could take notes and study them, which can be argued to elicit non-face perception strategies as DPs have the possibility of writing down specific image features to boost their memory. A task that allows for image-related recognition strategies (e.g., intentional learning and taking notes) does not correspond to the intention of the current study which aimed to measure true face perception/recognition processes. In sum, lots of gaps still exist in the present research on the perception of moving faces in DP, which were taken into account as much as possible in the current experiment (i.e., by implementing delayed recognition and incidental learning).

An incidental learning experiment combined with a delayed visual search paradigm was conducted to increase ecological validity and generalizability to real life face recognition. More specifically, in an initial incidental learning phase, faces were presented one at a time (and participants had to judge the friendliness of each face) and in a subsequent test phase participants had to identify the learned face in a series of three simultaneously presented faces. This design was partially adopted from Pilz et al. (2006), who suggested that this approach of incidental learning and “finding a friend in the crowd” parallels the real world and provides a behaviorally more relevant task than recognizing faces from a sequentially presented list. Moreover, such a design has not yet been implemented in a study with DPs and can thus potentially provide new insights in face recognition for this population. Between the learning phase and recognition phase, a retention period was furthermore implemented, consisting of general questions, thereby assessing delayed recognition instead of immediate recognition. During the learning phase, previously unfamiliar faces were presented as static images or as elastically moving faces (speech gestures) in full frontal view. The presentation-format of the faces during the recognition phase was either identical to the format during the learning phase or was switched (i.e., static in the learning phase and elastic in the recognition phase, or vice versa).

It was hypothesized that people with DP will experience a benefit from learning previously unfamiliar faces in elastic motion because of an enhanced representation of the structural form of the faces, thereby supporting the REH. Therefore, higher recognition rates were predicted for previously learned moving faces in the visual search array. In the context of the REH, a possible explanation for the motion benefit is potentially provided by the structural-reference hypothesis (Ganel and Goshen-Gottstein, 2004). This hypothesis posits that each face consists of first and second-order relations which give the face a unique structure. First-order relations refer to the arrangement of structural features in the face (e.g. two eyes above a nose), while second-order relations refer to the characteristic relative size of these first-order spatial relationships (Civile et al., 2016), such as two big blue eyes above a sharp nose. The movement of a face is limited and determined by its underlying structure, and as such, perception of movement provides additional information (i.e., more second-order information), hence potentially facilitating recognition. According to the REH, participants should identify the faces more accurately as the underlying 3D structure becomes more readily available when the face moves. Two experiments were conducted that shared an identical design. The aim of the first experiment was to obtain data from a group of normally developing young adults, to which subsequently collected data could be compared, and to replicate the beneficial effect of motion for unfamiliar faces. The second experiment was aimed at comparing the recognition scores of a DP group with age-matched controls, and at assessing whether the DP group also benefited from learning faces in motion.

## Methods

### Participants

In the first experiment, 18 subjects (eight male, 10 female) with an age range of 19–27 years (mean age 23.3, *SD* = 1.8) participated. In the second experiment, participants were divided into two groups: DPs and age-matched controls. Five prosopagnosics (one male, four female) with an age range of 33–52 years (mean age 43.4, *SD* = 9.4) and 10 age-matched controls (three male, seven female) with an age range of 40–55 years (mean age 48.5, *SD* = 6.5) participated.

Participants with self-reported face recognition skills who contacted the lab through an advertisement (*via* social media and personal contacts) were first invited for a semi-structured interview, which was adopted from Kennerknecht et al. (2006) and also used in other studies in our lab (Appendix A). During this interview questions were asked about face recognition, object recognition, social phobia, and other psycho-socio-biological perception abilities. In addition to the interview, the 20-item prosopagnosia index (PI20) was administered, a self-report instrument for DP (Shah et al., 2015). These two measures served as an initial screening to detect potential prosopagnosics and were supplemented by formal tests of face memory and perception, in confirmation with guidelines proposed by Dalrymple and Palermo (2016). More specifically, the Cambridge Face Memory Task (CFMT; Duchaine and Nakayama, 2006; Bowles et al., 2009) and the Cambridge Face Perception Task (CFPT; Duchaine et al., 2007; Bowles et al., 2009) were administered. Based on previous studies (e.g., DeGutis et al., 2012a,b), it was decided that a score of 46 (or below) on the CFMT and a score of 50 (or above) on the CFPT are indicative for the presence of DP. These formal tests were not administered to both groups. However, all participants qualitatively reported that they did not exhibit any facial recognition difficulties. Participants were assigned to one of the two groups based on their age alone.

### Materials

The experiment was created using PsychoPy v1.84.2 software (Pierce, 2007) and ran on an Acer Aspire E15 laptop equipped with an NVIDIA GEFORCE 940MX graphic card and a 1080 × 1920 full HD screen. During the learning phase, stimuli were between 8.2 and 12.3 cm tall, while the visual search arrays in the recognition phase consisted of three stimuli between 5.4 and 8.2 cm each. The participants were positioned approximately 45 cm in front of the screen, as such, the stimuli subtended a visual angle of approximately 6° horizontally and 4° vertically for the learning phase, and approximately 4° horizontally and 3° vertically in the recognition phase. Both static and dynamic face stimuli were obtained from Alice O’Toole’s moving faces database and consisted of non-moving full-frontal view faces and elastically (i.e., talking) moving faces (O’Toole et al., 2005). The faces were presented on screen as they would naturally occur (i.e., with hair visible, in color, and no manipulations to the natural variance of the face) in line with the suggestions made by Burton (2013). Non-Caucasian faces were excluded from the stimulus set because of the other-race effect, defined as poorer recognition of faces that observers are infrequently exposed to (e.g., other races), and its potential confounding influence on the data (e.g., Chiroro and Valentine, 1995; Michel et al., 2006; Anzures et al., 2013; Rivolta, 2014).

### Procedure

Each experiment followed an identical procedure and only differed in terms of participants. To make sure that all participants were naïve as to the real purpose of the experiment, the experiment was explained *via* an informed consent during which they were led to believe that they were participating in a study on the perception of friendliness. During this incidental learning phase, each face was presented for 10 s, after which the face disappeared and a Likert-scale ranging from one (very unfriendly) to seven (very friendly) emerged on the screen. Faces were presented for a fixed timeframe to ensure that every participant received the same amount of visual input and moreover, that the presentation time of static and moving faces was identical. Furthermore, although faces were presented in a random order, the presentation-format was systematically alternated between static and elastic. Participants rated a total of 24 faces on friendliness, from which 12 were presented as static full-frontal images and 12 as elastically moving videos. A short retention period was implemented during which participants had to answer six questions (e.g., “Do you know people with problems in recognizing faces?”), followed by an explanation of the real purpose of the study, and the recognition phase. Participants were told not to think too long about their responses but to try to be as accurate as possible during that phase.

**Figure 1:**
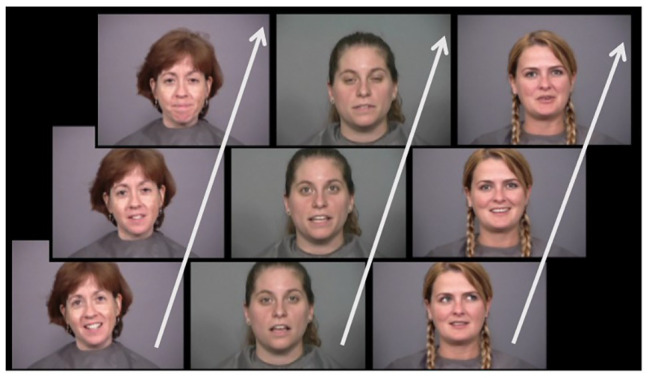
Example of a visual search array consisting of elastically moving faces. Note that the faces were presented as videos and not as image sequences.

During the recognition phase, faces were presented in a visual-search array consisting of three faces placed next to each other. The target faces were presented in the same format as during the learning phase, or were switched from static to elastic or vice versa, ultimately creating four different combinations and thus four levels of a within-subject independent variable: static versus static, static versus elastic, elastic versus static, and elastic versus elastic (see Figure 1 for an example of snapshots of a visual search array consisting of elastically moving faces). An ISI of 300 ms was implemented between a response and the next array. In total, 48 arrays were presented to the participant, of which 24 contained a target, and 24 contained only distractor faces. Participants pressed the spacebar if they could not recognize any of the test faces as the learned face, and had to press the left, middle, or right arrow to indicate the position of a recognized face.

### Statistical Analyses

To test the hypothesis of a potential beneficial effect of motion, hit rates and false alarm rates were first calculated for every condition and participant, which were further used to calculate d’ scores. Hit rates were calculated by dividing the number of correctly recognized faces by the total number of possible correctly recognized faces. False alarm rates were calculated by dividing the number of incorrectly recognized faces by the total number of possible correct rejections. d’ scores are then calculated by subtracting the Z-transform (based on the standard normal distribution) of false alarm rates from the Z-transform of hit rates. d’ is a parametric measure of sensitivity and a higher d’ score corresponds to better facial recognition. This procedure is more sensitive than an analysis, which is purely based on hit rates, as it includes an indication of the accept/reject criterion adopted by individual participants. Second, separate bias-corrected and accelerated (BCa) nonparametric bootstrapped paired-samples *t*-tests were performed to analyze possible differences between the conditions within each group. Bootstrapping was applied because of the small sample sizes in each experiment and because it is less dependent on normal and symmetrical sampling procedures, while BCa was applied to improve standard confidence intervals (DiCiccio and Efron, 1996; Preacher et al., 2007). Third, a nonparametric bootstrapped independent-samples *t*-test was performed to analyze possible differences between the DP group and the age-matched controls. Finally, separate nonparametric bootstrapped paired-samples *t*-tests were performed on the hit rates and false alarm rates within each group to analyze whether there was a potential difference between total number of hit rates and false alarm rates. With each analysis, the original sample was replicated 2,000 times and a 95% confidence interval and an alpha level of 0.05 were used.

## Results

### Analysis I: Experiment 1

**Figure 2:**
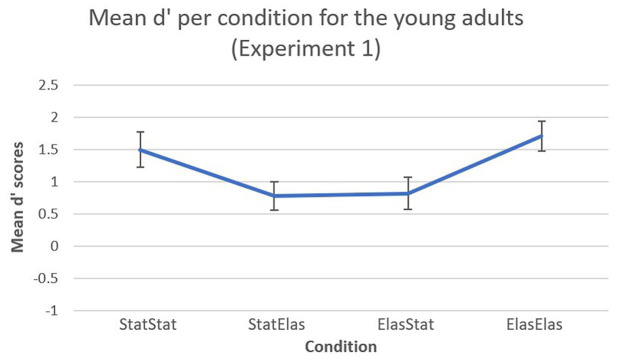
Mean d’ recognition scores (and error bars) per condition. In the static-static and elastic-elastic conditions, participants showed higher sensitivity in detecting target faces compared to the other conditions.

A visual representation of the mean d’ scores (including error bars) in Experiment 1 is shown in Figure 2. For the young adults in Experiment 1, the bootstrapped paired-samples *t*-test revealed significantly higher mean d’ recognition rates for the elastic-elastic condition (*M* = 1.7030, *SD* = 0.9828) compared to the static-elastic (*M* = 0.7726, *SD* = 0.9479) and elastic-static (*M* = 0.8159, *SD* = 1.0566) conditions [*p* = 0.019, 95% CI (0.2830, 1.4942), and *p* = 0.004, 95% CI (0.4464, 1.3550), respectively). Furthermore, the static-static condition (*M* = 1.4948, *SD* = 1.17) elicited significantly higher mean d’ recognition rates compared to the static-elastic condition [*p* = 0.034, 95% CI (0.1715, 1.2627)]. The static-static condition also elicited higher mean d’ recognition scores compared to the elastic-static condition, however, this difference was not significant [*p* = 0.063, 95% CI (0.1964, 1.2438)]. Almost no difference could be observed in mean d’ recognition scores between the elastic-static and the static-elastic conditions [*p* = 0.900, 95% CI (−0.6874, 0.6106)]. Finally, almost no differences were observed between the static-static and elastic-elastic condition [*p* = 0.508, 95% CI (−0.7646, 0.4115)].

### Analysis II: Developmental Prosopagnosia and Age-Matched Controls (Experiment II)

**Figure 3:**
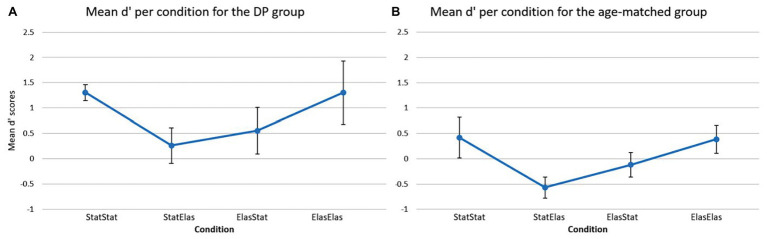
Mean d’ scores (and error bars) per condition for the DP group **(A)** and for the age-matched controls **(B)**. The DP group showed higher sensitivity rates in all conditions compared to the age-matched group. The age-matched group showed the lowest sensitivity in the static-elastic condition and similar sensitivity rates in the static-static and elastic-elastic conditions. A similar profile can be detected in the DP group.

Figure 3 depicts the mean d’ score in each of the four conditions for the DP group (Figure 3A) and the age-matched controls (Figure 3B). For the DP group, the bootstrapped paired-samples *t*-test revealed no significant differences in mean d’ recognition rates between the conditions. The static-static condition (*M* = 1.3059, *SD* = 0.3587) showed no difference with the elastic-elastic condition [*M* = 1.3059, *SD* = 0.6263; *p* = 1.000, 95% CI (−0.2796, 0.3008)]. Slightly higher (but not significantly different) mean recognition rates were observed in the static-static condition compared to both the static-elastic (*M* = 0.2615, *SD* = 0.7792) and elastic-static (*M* = 0.5532, *SD* = 1.021) condition [*p* = 0.095, 95% CI (0.3160, 1.8781), and *p* = 0.129, 95% CI (0.1814, 1.2410), respectively]. A slight (but again not significant) advantage of the elastic-elastic condition compared to the static-elastic and elastic-static conditions was furthermore observed [*p* = 0.079, 95% CI (0.5094, 1.5388), and *p* = 0.067, 95% CI (0.4792, 1.1094), respectively]. Finally, the elastic-static condition showed slightly higher mean d’ recognition rates compared to the static-elastic condition [*p* = 0.587, 95% CI (−0.9523, 0.4580)].

For the age-matched group, Figure 3B shows a visual representation of the results. The bootstrapped paired-samples *t*-test only revealed slightly higher (but not significant) mean d’ recognition rates for the elastic-elastic condition (*M* = 0.3855, *SD* = 0.8736) compared to the static-elastic (*M* = −0.5699, *SD* = 0.6705) condition [*p* = 0.082, 95% CI (0.2705, 1.6618)]. No differences were found between the static-static (*M* = 0.4194, *SD* = 1.2695) and elastic-elastic condition [*p* = 0.935, 95% CI (−0.9456, 1.0744)]. Furthermore, the static-static condition only showed slightly higher (but not significant) mean d’ recognition rates compared to the elastic-static (*M* = −1.2014, *SD* = 0.7695) and static-elastic conditions [*p* = 0.204, 95% CI (−0.1331, 1.2645), and *p* = 0.099, 95% CI (−0.0401, 2.005), respectively]. Finally, the elastic-static condition showed slightly lower (but again not significant) mean d’ recognition rates compared to the elastic-elastic [*p* = 0.251, 95% CI (−0.4361, 1.2666)] and static-elastic [*p* = 0.194, 95% CI (−1.0034, 0.0541)] conditions.

Based on the visual representation of the data, it seems somewhat striking that the DP participants showed overall higher mean d’ recognition scores in comparison to the age-matched controls. This was further explored in a bootstrapped independent-samples *t*-test which analyzed each condition separately and compared the two groups directly. This analysis indeed revealed that DPs (*M* = 1.3059, *SD* = 0.6263) performed significantly better in the elastic-elastic condition compared to the age-matched group (*M* = 0.3855, *SD* = 0.8736) group [*p* = 0.036, 95% CI (−1.6822, −0.0694)]. Furthermore, DPs performed marginally better on the static-static condition compared to the age-matched group (*M* = 1.3059, *SD* = 0.3587, and *M* = 0.4194, *SD* = 1.2695, respectively), but this result was not significant [*p* = 0.096, 95% CI (−1.7151, −0.0782)]. Likewise, a marginally, but not significant, better result could be observed in the static-elastic condition [*p* = 0.063, 95% CI (−1.5519, 0.0069)] for the DP group compared to their age-matched counterparts (*M* = 0.2615, *SD* = 0.7792, and *M* = −0.5699, *SD* = 0.6705, respectively). Finally, DPs performed slightly better compared to the age-matched controls in the elastic-static condition (*M* = 0.5532, *SD* = 1.021, and *M* = −1.2014, *SD* = 0.7695, respectively), however, this difference was not significant [*p* = 0.212, 95% CI (−1.4932, 0.2782)]. These surprising findings are further deliberated in the discussion section.

### Analysis III: Additional Analysis on Hit Rates and False Alarm Rates

**Figure 4:**
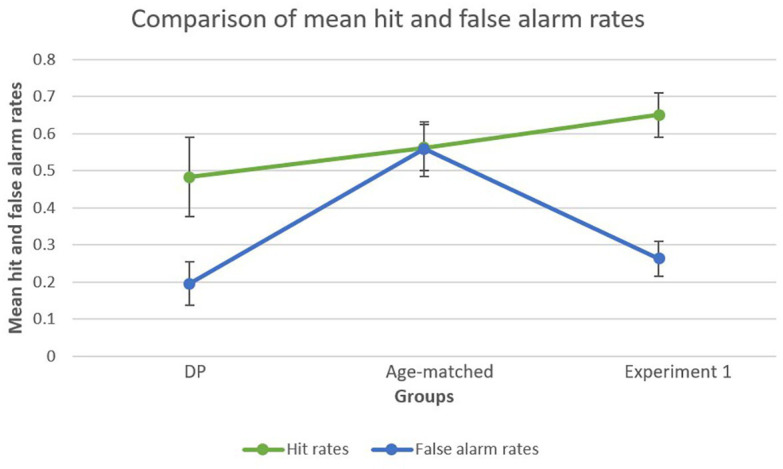
Mean false alarm rates and mean hit rates within each group. Both the DP group and the young adult group (Experiment 1) showed a marked difference between mean hit rates and mean false alarm rates, whereas the age-matched group showed similar hit and false alarm rates.

To further explore the striking difference in d’ scores between the age-matched control group and DP group, further analyses were conducted on hit rates and false alarm rates separately. This analysis was also performed on the young adults of Experiment 1 in order to create a general overview. A bootstrapped paired-samples *t*-test performed for the DP group revealed a significant difference between mean hit rates (*M* = 0.4833, *SD* = 0.2397) and mean false alarm rates (*M* = 0.1958, *SD* = 0.1332), showing higher hit rates compared to false alarms [*p* = 0.003, 95% CI (0.1542, 0.4000)]. In the age-matched control group, this analysis did not reveal a significant difference [*p* = 0.101, 95% CI (−0.0210, 0.2876)] between mean hit rates (*M* = 0.5625, *SD* = 0.1977) and false alarm rates (*M* = 0.5583, *SD* = 0.2328). Finally, for Experiment 1 the analysis revealed a significant difference between mean hit rates (*M* = 0.6505, *SD* = 0.2528) and false alarm rates (*M* = 0.2627, *SD* = 0.0234) in favor of the former [*p* < 0.001, 95% CI (0.3333, 0.6169)], a similar finding as observed in the DP group. Figure 4 provides a visual representation of the results.

## Discussion

In the present study, participants incidentally learned static and elastically moving faces, which were to be recognized in visual search arrays during a delayed recognition phase. Movement and incidental learning were key factors in the design, under the assumption that these factors support a more ecologically valid approach and thereby generalizability to everyday life. During the recognition phase, learned faces could either keep their initial presentation (i.e., static or elastic) or switch in presentation, ultimately creating four within-subject conditions. It was hypothesized that when incidentally learning an unfamiliar face presented in elastic motion, DPs will show higher sensitivity to identify and recognize that face in a delayed visual search array. As the faces were unfamiliar, and knowledge of idiosyncratic movements can be argued to be limited (faces were only presented for 10 s in the learning phase), it was hypothesized that DPs would benefit from viewing the face in motion given that more views (i.e., more frames per second) can potentially enhance the 3D structure of that face in memory. Such findings could provide evidence for the REH.

In a first experiment, sensitivity of detecting and identifying the target face was measured in a group of young normally developing adults. Significantly higher mean d’ recognition rates for the elastic-elastic condition compared to the static-elastic and elastic-static conditions were observed. It seems that faces learned in motion provide higher sensitivity to recognition, however only when these faces are presented in motion during recognition as well. The static-static condition also elicited significantly higher mean d’ recognition rates compared to the static-elastic condition. Looking at the results for the elastic-static condition alone, evidence in terms of a beneficial effect of motion cannot be reported. Although the elastic-elastic condition differed significantly from the elastic-static and static-elastic conditions, it only slightly differed from the static-static condition. It appears that faces, which are presented in the same format during learning and recognition, are better recognized compared to faces that are presented in a different format. Taken together, it must be concluded that, in this first experiment with young adults compelling evidence for the REH cannot be provided and thus findings of previous experiments that did find such evidence are not replicated in the current study.

The second experiment was intended to measure the sensitivity of detecting and identifying the target face in a group of DPs, compared to age-matched controls. A beneficial effect of motion could not be detected in either of the groups and thus evidence for the REH cannot be formulated. The DPs showed the same sensitivity for the static-static and elastic-elastic condition, and even though these two conditions showed a trend toward better sensitivity as compared to the static-elastic and elastic-static conditions, the differences were not significant. The elastic-static condition did show a slight advantage relative to the static-elastic condition, however, this is probably due to natural variation and is by no means systematic nor significant. The age-matched controls also showed similar sensitivity rates for the static-static and elastic-elastic conditions. DP participants showed significantly higher sensitivity in the elastic-elastic condition compared to their age-matched counterparts. This is surprising as it would rather be expected that the typically developed age-matched people would show better results. This strange result might be explained by the tendency of the age-matched controls to exhibit higher false alarm rates, thereby lowering their composite d’ score. DPs, in contrast, tended to less frequently recognize faces falsely, thereby not affecting their composite d’ scores. This potential explanation is further elucidated below. Finally, DPs also showed higher sensitivity in all other conditions, although these results were not significant.

In sum, participants did not seem to benefit from incidentally learning elastically moving faces, and thus, did not seem to notice the enhanced 3D structure of the faces that could have resulted from this motion. It appears moreover, that the sample tested in the current study showed a contra-intuitive result as the age-matched controls seemed to perform worse than the DPs. The low performance of the age-matched control group compared to the young adult group of Experiment 1 could potentially be explained by a decline in face recognition performance related to aging, as supported by previous research (e.g., Adams-Price, 1992; Lamont et al., 2005; Boutet and Faubert, 2006). However, as these control participants were matched on age with the DP group, it would be more intuitive to expect that they would still be better in recognizing faces than same-aged people who are impaired in face recognition.

Another, and possibly more acceptable explanation, can be formulated based on the differences between hit and false alarm rates within each group. In the current study, it seems that both DPs and the young adult group showed significantly higher hit rates compared to false alarm rates while age-matched controls showed no difference between these rates. As d’ scores are calculated by subtracting the false alarm rates from the hit rates, this might possibly have been the reason for the lower d’ scores observed in the age-matched group. More false alarms result in a lower total d’ score. These results do not necessarily imply that the age-matched controls performed worse on this task. It is perfectly possible that they had a very high hit rate (thus recognizing a lot of the previously learned faces). However, for some (yet unclear) reason, they simultaneously recognized several faces falsely, thereby possibly concealing their true capacities. As the current experimental design did not allow to investigating this tendency in more depth, future research is warranted to keep these possibilities in mind. In sum, these observations might indicate that DPs complete such recognition tasks in a qualitatively different way than their age-matched counterparts do. This is an interesting route to explore in future research.

Some general criticisms can be formulated concerning the current study and may additionally explain why the observed effects are mostly not significant. First, and most importantly, taking the rather small sample size of the current study into consideration (i.e., 10 age-matched controls and five DPs in Experiment 2), it is possible that insufficient participants were involved to reach the average population performance on tests of face recognition. A small sample size might increase the possibility of assuming a false premise to be true (Faber and Fonseca, 2014). In the current study, the sample size might have been too small to reflect a true population average. A larger sample size is advised. Very small samples may furthermore undermine the internal and external validity of a study (Faber and Fonseca, 2014), and therefore the results of the current study should be interpreted with caution. This criticism applies to other research on DP and dynamic face recognition as well. Therefore, all researchers who want to explore this field are advised to do a proper power analysis prior to the study and, although this may not be straightforward, implement a larger sample of DPs to be tested.

Second, the present design may be confounded by picture-matching instead of face-matching mechanisms as the static-static and elastic-elastic conditions showed overall better recognition compared to the other conditions. It is commonly observed in recognition research that a direct link exists between the similarity of images presented during learning and test-phase, and the recognition rate (e.g., Ritchey et al., 2013; Chen et al., 2015). Several previous studies (e.g., Lander et al., 2004; Pilz et al., 2006) have implemented changes in viewpoint or expression during recognition to ensure that they were assessing face processing instead of identical picture matching (i.e., same background lighting, same shutter settings, same focal length, et cetera), which could serve as an alternative strategy to recognition. Furthermore, it is argued that unfamiliar face recognition is more heavily influenced by image-level characteristics (Hancock et al., 2000), which is particularly relevant for the current study as unfamiliar faces were used in the design. However, even if a design had been created with changes in viewpoints of the static faces, possibly detecting a motion-advantage for the moving faces could still have resulted from worse recognition of the static faces due to added difficulty of presenting static faces in a different viewpoint, especially in the case of unfamiliar faces (Hancock et al., 2000). Bruce (1982) has for example, shown that the hit rates of face recognition dropped significantly when expression and viewpoint were changed at test. For this reason, pictures and videos were not altered or presented differently during the recognition phase to avoid the possible static-face-disadvantage effect. Additionally, opting for changes in viewpoints could have made the experiment too complicated, especially for the DP group. Nevertheless, picture-matching and potentially even video-matching could indeed be a likely explanation for the better recognition rates found in the static-static and elastic-elastic conditions. Future studies should explore these issues more in depth and should subsequently implement viable solutions when adopting the current design.

Third, another potential criticism is the inclusion of some faces that exhibited emotions. Emotions may influence face perception and later recognition, as demonstrated by previous research (e.g., D’Argembeau et al., 2003; Huis in’t Veld et al., 2012). In addition, several researchers have reported worse emotion recognition in DP compared to normal controls (e.g., Biotti and Cook, 2016), but studies reporting intact facial emotion recognition in DP exist as well (e.g., Duchaine et al., 2003; Humphreys et al., 2006). There is no information on potential difficulties with emotion recognition for the DPs as this was not evaluated prior to the study. Taken together, it could have been possible that some faces in certain conditions exhibited emotion expressions, which were potentially easier to be transferred to memory and may have confounded later sensitivity to recognition. Therefore, future studies adopting the current design should be more cautious and eliminate emotion expressions in their dataset.

Fourth, Longmore and Tree (2013) reported that their task may have been too challenging for DPs which could have made it less likely to find a beneficial effect of motion. In their study, participants had to learn a number of faces during a limited exposure time (i.e., each face was presented for only 6 s during learning). It can be argued that this was also the case for the present design, as participants were exposed to 24 novel faces, which were presented for only 10 s. Moreover, because the learning phase was incidental, participants were limited in the possibility of intentionally memorizing some characteristic facial information. However, since the learning phase consisted of rating the faces in terms of their perceived friendliness, it could be argued that their attention was selectively focused on the faces and that they were processing the visual input more in depth than when merely presenting the faces without rating for perceived friendliness. Nonetheless, it is difficult to argue against the hypothesis that the task may have been too complex and the challenges it may have imposed on both the DP group and the age-matched controls. Future designs of the sort described here could potentially benefit from an extended period of learning each face and reducing the number of faces overall.

Finally, some researchers have suggested a distinction between the perception of familiar and non-familiar faces in terms of the beneficial effect of motion, mostly in favor of familiar face processing (e.g., Christie and Bruce, 1998; Bruce et al., 1999; Lander and Chuang, 2005), although other researchers also found a beneficial effect of motion for unfamiliar faces (e.g., Pike et al., 1997; Thornton and Kourtzi, 2002). A study on face recognition in surveillance videos found that familiar faces were still recognized even when the faces were obscured, whereas unfamiliar faces were as poorly recognized by participants without experience in forensic identification as more experienced participants (Burton et al., 1999). This suggests that observers are generally quite bad at recognizing unfamiliar faces. In the context of familiar faces, these observers have already stored ample exemplars in memory as the face is encountered in a multitude of instances characterized by a wide range of viewpoint variations, lighting, expression, et cetera. This makes it more likely that these stored representations will be generalized to novel instances such as variation in viewpoints (Christie and Bruce, 1998). A face in motion is characterized by dynamic changes in viewpoints, and consequently observers could connect these different representations with the representations already stored in memory, hence aiding rapid recognition of that face. This mechanism is probably less beneficial in the context of unfamiliar faces as these faces are never seen before and hence less stored representations exist for these faces in memory to aid recognition. As motion can potentially enhance perception of the 3D structure and thus representation of a face, it could be argued that familiar faces benefit more from this enhancement than unfamiliar faces. This could possibly be the reason for the lack of a beneficial effect of motion in the current study.

Even though the current design is clearly far from perfect, some interesting markers were identified for potential future studies in the realm of face recognition research. Given that none of the studies on DP and moving faces described in the present article have focused on incidental learning, it is possible that the results described here form an interesting indication for future research to directly compare incidental and intentional learning tasks in this population. Findings of such comparisons might prove valuable for implementation in both the SIH and REH. It is possible that participants who expect a memory test may emphasize the encoding of information that they believe is most appropriate for the task (Neill et al., 1990). Consequently, a beneficial effect of motion should be easier to detect in an intentional learning task as participants may view the characteristic motions and 3D structure of the face to be the most appropriate cues for later memory retrieval. In contrast, during an incidental learning task, participants may not elaborate on these cues simply because of not expecting them to be profitable. These hypotheses may provide an interesting subject for future research.

In conclusion, a beneficial effect of elastic motion for DPs following an incidental learning task could not be replicated, and moreover, the current study did not provide evidence for the REH. A surprising observation in the current study was the overall low sensitivity rate for the age-matched control group compared to the DP group. A more thorough examination of d’ scores showed that the age-matched control group recognized as many faces correctly as incorrectly, while DPs recognized significantly more faces correctly compared to falsely recognizing faces. Future research is warranted to keep these findings in mind and try to replicate them in a larger sample. The criticisms stated above are hard to change when one is primarily interested in reflecting real life as much as possible (i.e., ecological validity), as a trade-off inherently exists between ecological validity and experimental control. In the current study, ecological validity was increased as much as possible, however, this came with the cost of somewhat weaker experimental control. Future research adopting the current design and objective is especially warranted to test a larger sample as strong statistical power makes it more probable to detect a beneficial effect of motion despite the potential noise that will inevitably seep through the pores of the design. Finally, researchers should be cautious in creating an experiment that is unnecessarily complex and too challenging, as this complexity may be a pitfall concerning the possibility of finding results that are in accordance with real-life face recognition.

## Data Availability Statement

The datasets generated for this study are available on request to the corresponding author.

## Ethics Statement

Written informed consent was obtained from the individuals for the publication of any potentially identifiable images or data included in this article.

## Author Contributions

All authors contributed to framing the study theoretically, designing and executing the experiments, analyzing the data, interpreting the results, and drawing conclusions. All authors contributed to the article and approved the submitted version.

### Conflict of Interest

The authors declare that the research was conducted in the absence of any commercial or financial relationships that could be construed as a potential conflict of interest.
