# Supplementary material for: Developmental Prosopagnosia and Elastic Versus Static Face Recognition in an Incidental Learning Task
Source: Front Psychol. 2020 Aug 31;11:2098. doi: 10.3389/fpsyg.2020.02098 (PMC7488957; doi:10.3389/fpsyg.2020.02098)
Supplement: Supplementary file 1 [file Table_1.DOCX]

**Semi-Structured Interview for Congenital PA**

| Name: |
| --- |
| First name: |
| Date of birth: |
| Profession: |
| E-mail: |
| Left/right handed: |

**1. Introduction**

Onset of problems?

History of diseases of interviewed participant?

Eyes (sight) and other senses.

Learning disabilities? (reading, arithmetic, writing)

**2. Social behaviour**

In group

With familiar versus unfamiliar people

**3. Sense of orientation.**

For example: in a foreign city, with or without map?

**4. Object recognition.**

a. Animals.

Do you have domestic animals? Can you differentiate between dogs and cats? And birds?

Mental imagery of animals:

O In detail, 3D, vivid O like a picture O in colours/black white O vague

b. Trees

Able to disriminate between different sorts of trees?

Mental imagery of trees :

O In detail, 3D, vivid O like a picture O in colours/black white O vague

Mental imagery of a large, beautifully red, thorny rose:

O In detail, 3D, vivid O like a picture O in colours/black white O vague

c. Space

Visual walk about, e.g. in one’s own living room. Where is everything located? Mental imagery or only from memory?

Able to rotate a 3D object mentally?

d. Other objects

Able to find one’s own coat in a pile of coats? Or mobile phone?

**5. Face perception**

a. Estimate age based on face? Yes/No

b. Estimate gender based on face? Yes/No

c. Reading emotions from a face? Yes/No

d. Score attractiveness of a face? Yes/No

e. Eye contact during conversation? Yes/No

If not, where do you focus?

f. Does it strike you when somebody else is looking at you?

g. Mental imagery of:

Good friend

O In detail, 3D, vivid O like a picture O in colours/black white O vague

Parents

O In detail, 3D, vivid O like a picture O in colours/black white O vague

Interviewer (close the eyes)

O In detail, 3D, vivid O like a picture O in colours/black white O vague

**6. Face recognition**

How do you recognize somebody who is sitting on a bench (somewhat at a distance)? On the basis of the face or another source of information?

How do you recognize somebody who is walking towards you?

How do you recognize somebody in a social context (parties,…)?

How long do you need to observe somebody in order to recognize him or her later that day/a week later?

How long does it take you to recognize somebody, on the basis of the face, who participates in a large group of similarly looking people?

Did it occur to you that you were unable to recognize somebody who is familiar to you?
O never O seldom O sometimes O relatively often O often

What is your reaction in that situation?

Did you ever pass somebody that you know well/vaguely?

Do you avoid situations in which you are supposed to recognize others?

Are you the first to greet people or vice versa?

Once you are greeted, are you then able to recognize that person?

Do people sometimes judge you as arrogant or unfriendly? If so, what is the underlying basis according to your opinion?

Do you sometimes experience difficulties to recognize somebody solely on the basis of the face (and therefore not only remember his or her name)?

When you are watching a movie, do you succeed to follow the actors? And what happens in movies in which they always wear the same outfit or are very similar to each other? On the basis of what features do you follow the line of story?

Do you recognize friends in disguise, like during Halloween?

If you would tumble across a famous actor/singer/politician, would you recognize him/her?

Suppose you are waiting for somebody at the airport or the railway station and you know that person only from a picture, would you succeed to recognize him/her?

Suppose you walk into a crowded restaurant and your friends/family members/colleagues already went to table. Would you be able to find them?

Do you have difficulties recognizing family members or yourself on the basis of old photographs?

**7. Miscelaneous**

a. Do you have family members with a similar problem?

b. When did you notice that face recognition is more taxing for you than for others?
